# Managing Dystonia in Partington Syndrome

**DOI:** 10.1002/mdc3.70356

**Published:** 2025-09-30

**Authors:** Emilie Pichon, Aurea Alioth, Sabina Catalano Chiuvé, André Zacharia, Marta Ruiz‐Lopez, David P. Breen, Florence Zangas‐Gehri, Florent Draye, Aurore Curie, Duncan Wilson, Victor S.C. Fung, Stephen Duma, Joël Fluss, Julien F. Bally

**Affiliations:** ^1^ Service of Neurology, Department of Clinical Neurosciences Lausanne University Hospital (CHUV) and University of Lausanne (UNIL) Lausanne Switzerland; ^2^ Department of Neurology University of Geneva and University Hospitals of Geneva Geneva Switzerland; ^3^ Movement Disorder Neurology University Hospitals Cruces Bilbao Spain; ^4^ Neurodegenerative Diseases Group Biocruces Bizkaia Health Research Institute Barakaldo Bizkaia Spain; ^5^ Anne Rowling Regenerative Neurology Clinic, Institute for Neuroscience and Cardiovascular Research University of Edinburgh Edinburgh; ^6^ Pediatric Neurology Unit, Subspecialities service, Department of Pediatrics University of Geneva and University Hospitals of Geneva Geneva Switzerland; ^7^ Institute of Neuroinformatics ETH Zurich and University of Zurich Zurich Switzerland; ^8^ Reference Center for Intellectual Disability from Rare Causes, Child Neurology Department, Woman Mother and Child Hospital Hospices Civils de Lyon, F‐69500 Bron France; ^9^ Lyon Neuroscience Research Centre CNRS UMR5292, INSERM U1028, F‐69500 Bron France; ^10^ Lyon University F‐69008 Lyon France; ^11^ Edmond J. Safra Movement Disorder Fellow, Westmead Hospital Sydney Australia; ^12^ Movement Disorders Unit Department of Neurology, Westmead Hospital and Sydney Medical School University of Sydney Sydney Australia; ^13^ Department of Neurology, St George Hospital Sydney Australia; ^14^ St George and Sutherland Clinical School UNSW Medicine Sydney Australia

**Keywords:** dystonia, treatment, Partington, Aristaless‐related homeobox (*ARX*) gene, “Geste antagoniste”

## Abstract

**Background:**

Bilateral focal hand dystonia is an almost pathognomonic sign of Partington syndrome, frequently accompanied by intellectual disability and oromotor dyspraxia. However, a few studies have focused on the treatment of this focal dystonia, making patient management uncertain.

**Cases:**

We present 2 cases of Partington syndrome featuring Aristaless‐related homeobox (ARX) gene mutations, hand dystonia, and other clinical signs. Various drug treatments were attempted, including levodopa (l‐dopa), trihexyphenidyl, tetrabenazine, and benzodiazepines, as well as botulinum toxin. Additionally, a blinded dystonia protocol was used to assess l‐dopa's efficacy in 1 patient, which confirmed only mild benefit.

**Literature review:**

Through a systematic review of the literature, we found that only l‐dopa and baclofen might result in mild improvement, whereas propranolol, gabapentin, and haloperidol were reported as ineffective. The descriptions in those studies were, however, imprecise and the improvement rather mild, hindering definitive conclusions about their effectiveness.

**Conclusions:**

Treatment options in Partington syndrome–associated dystonia remain elusive. Further research and additional case studies are needed to fully characterize the clinical features of Partington syndrome and to identify effective treatments.

Partington syndrome is an X‐linked recessive developmental disorder that was first identified in 1988 through the detailed description of a family of 10 men characterized mainly by intellectual disability, episodic and nonprogressive dystonic hand movement and dysarthria.^1^ In 2002, the disease was subsequently described as part of the spectrum of diseases linked to a mutation in the *Aristaless‐related homeobox* (*ARX*) gene, itself located on the short arm of the X chromosome (Xp22.13).^2^ The most frequent mutation is a duplication of 24 bp in exon 2 (c.429_452dup24), which leads to the expansion of the second polyalanine tract from 12 to 20 alanines and can result in multiple phenotypes within the same family.^3^ This ranges from West syndrome,^4^ the first clinical syndrome found linked to the *ARX* gene and characterized by infantile spasms with hypsarrhythmia and mental retardation, to Partington syndrome.^2^

The distinctive and characteristic, almost pathognomonic, clinical manifestation of Partington syndrome is a focal dystonia affecting the hands and wrists, often elicited by asking the individual to hold a pen or pencil.^5^, ^6^ Dystonia can even impact the face and muscles involved in articulation, leading to dysarthria, and has been observed in some cases to affect the feet,^7^, ^8^ Curie et al.^9^ completed the definition of the phenotypic expression of the mutation (c.429_452dup24), and the term “Partington syndrome” was defined to characterize all patients with this mutation who present with motor deficits, ranging from kinetic apraxia to focal dystonia. However, there remains a lack of reliable information in the literature regarding the treatment of the motor disorders in Partington syndrome, particularly hand dystonia.

To better understand the management of dystonia in Partington syndrome, we conducted a systematic review and provided a detailed description of 2 new cases (with accompanying videos) presenting an *ARX* pathogenic variant with mild intellectual disability and hand dystonia. For those cases, we describe the different therapeutic approaches tried for managing dystonia, including a blinded trial with levodopa (l‐dopa).

## Case Series

### Case 1

An 18‐year‐old right‐handed man, second in a family of 4 siblings, was born healthy at 39 weeks via vaginal delivery. From early childhood, he exhibited language and motor delays. At 8 years old, his speech became intelligible, but he still presented a severe developmental speech and language disorder (in syntax, lexical, and phonological domains). He struggled with the alphabet, counting, and visuospatial tasks, and suffered from attentional disorder. Fine motor skills like writing and drawing also remained challenging. He exhibited gestural dyspraxia, more pronounced on the left; an immature pen grasp; bucco‐linguo‐facial apraxia; and intermittent drooling worsened by stress. He received specialized education with speech and occupational therapy. A methylphenidate trial was stopped due to depressive behavior.

At age 14, a neuropsychological assessment revealed cognitive improvements; however, a mild intellectual disability was still present. Difficulties also persisted in language, oromotor functions, and gestural dyspraxia. In addition, abnormal right‐hand posturing described as dystonic appeared, with involuntary wrist flexion at rest, disappearing during movement but accompanied by pain. The dystonia also affected his left side, limiting thumb–index opposition, though with less impact. Partington syndrome was suspected, and the diagnosis was confirmed by genetic analysis (c.430_453dup24 mutation in the *ARX* gene). Magnetic resonance imaging (MRI) was not performed. By age 16, motor deficits were stable or slightly improved, but dystonia and fatigue were the most limiting symptoms. At age 18, reevaluation showed slight dystonia progression, with more pronounced left‐side thumb–index opposition inability.

During the physical examination at age 18, he was alert and cooperative, with no facial dysmorphia. A nasopalpebral reflex persisted without grasping. Dystonia in the hands made the assessment of gestural praxis challenging. Jaw dystonia, along with bucco‐linguo‐facial apraxia, was also present. Writing was readable despite abnormal pen holding between the lateral sides of the middle and fourth fingers and the thumb's proximal phalanx. The left wrist exhibited 100° flexion and ulnar deviation, rendering it nearly unusable. A dystonic tremor, more pronounced on the left, and overflow dystonia during walking or running were observed, whereas no dystonic posture in the legs was noted.

l‐Dopa was tried at the age 18 years, and clinical response was assessed using a blinded protocol. Results (Appendix A: part A: blinded assessment by 3 independent movement disorders experts from 3 movement disorders centers [United Kingdom, Spain, and Switzerland]) showed mild improvement in dystonia severity at 800 mg per day compared to no l‐dopa, based on the (BFM) Burke‐Fahn‐Marsden severity scale severity scale. Differentiating between the effects of 400‐ and 800‐mg doses based on BFM severity scale was less clear. Overall, experts agreed that the video (Video 1) without treatment showed the worst condition overall, whereas the video (Video 2) at 800 mg was the best and 400 mg was intermediate (Video 3), with an excellent interrater agreement. The patient reported subjective improvements, including reduced sweating and better ability to open the hand at night, but he perceived these improvements as mild and eventually tapered off l‐dopa due to the burden of taking 4 pills daily. The patient then reported increased palmar sweating, which he himself attributed to more clenched hands after l‐dopa discontinuation.

**Video 1:** The initial examination reveals facial and bilateral hand dystonia, more pronounced in the left hand. Activities like finger‐to‐thumb opposition are particularly challenging. Note the distinctive pen grip observed during writing.

**Video 2:** After 2 months on a stable dose of 400 mg per day of levodopa, there is a mild improvement, manifesting as a reduction in tremor in the left hand.

**Video 3:** After 3 months on a stable dose of 800 mg per day of levodopa, there is mild but significant improvement. Stabilization of hand movements and better hand opening are observed.

Neuropsychological assessments (Appendix A: part B: unblinded assessment by our neuropsychologist at baseline and then at 800‐mg l‐dopa per day) revealed persistent language and manual dexterity impairments, particularly in the left hand. The dystonic postures and movements of the hands make interpreting gestural praxis difficult, which nevertheless seems preserved. At 800‐mg l‐dopa, manual dexterity slightly decreased in the right hand but improved in the left. Bucco‐linguo‐facial praxis improved, along with alternating gestures performed posttreatment. Copying the Rey figure showed qualitative and quantitative improvement, possibly due to a training effect.

During basketball, the patient's hand opened before the ball touched it, likely aided by visual/auditory cues (Video 4).

**Video 4:** This video demonstrates the patient playing basketball. Note the opening of the left hand before the basketball's arrival. We hypothesize this could be due to an auditory and/or visual sensory trick.

### Case 2

A 27‐year‐old man with 2 healthy sisters has a maternal family history of *ARX* mutation, including an uncle with intellectual delay, seizures, and generalized dystonia but with milder dystonic posturing of the hand and minor dysmorphic features. He is also the only affected sibling, and no similar symptoms have been reported in other earlier generations. There were no pregnancy complications, but the patient was born hypotonic and initially diagnosed with cerebral palsy. Motor development was atypical; he failed to crawl and instead moved by bottom shuffling. The walking became optimal only at the age of 8 when he also started speech development. He maintained good hand skills despite early hand dystonia. His dystonia, initially presenting as clenched fists, later progressed to painful dystonia in the wrist and finger joints. Pain worsens with activity and was often accompanied by joint swelling, complicating tasks like tying shoelaces or fastening buttons. In addition to hand symptoms, he experienced worsening bruxism due to jaw dystonia, which caused him to clench and grind his teeth.

He received specialized education and transitioned to mainstream high school, completing the 12th grade. At age 17, genetic testing confirmed a 24‐bp duplication in the *ARX* gene, consistent with the clinical phenotype of Partington syndrome (reports lack genotype specification). His mother is a carrier. Complementary tests, including hand and wrist radiographs, brain and cervical spine MRI, electroencephalogram, and an autoimmune panel, were normal. Neurophysiology showed left median neuropathy, with borderline findings on the right.

During the physical examination (Video 5), the patient appeared jovial but became withdrawn and embarrassed. He presented with intellectual disability, a narrow face, a high arched palate, a single palmar crease, and inverted nipples. Eye movements showed saccadic pursuit and oculomotor apraxia. Muscle tone and strength were normal. Upper‐limb movements were slow with reduced amplitude and intermittent bilateral tremor. He was unable to perform fractionated finger movements, likely due to limb‐kinetic apraxia beyond dystonic posturing. Joints were painful on palpation, and dystonia affected toes and feet during walking. Reflexes were absent in the upper limbs but normal in the lower limbs. Mild tandem walking impairment was noted, without clear ataxia.

**Video 5:** This examination highlights limb‐kinetic apraxia in the upper limbs. No resting dystonia is observed; however, during attempted hand movements, clear dystonia results in finger flexion in both hands. The same distinctive hand grip as in case 1 is observed during writing.

Therapeutic strategies included managing dystonia and pain. Trihexyphenidyl was first tried when he was 26 years old, starting at 2 mg per day (duration unknown) and then increased to 4 mg per day for 2 months (up to 2 mg twice daily). It did not improve either the pain or the dystonia and was discontinued due to drowsiness and urinary issues. l‐Dopa 300 mg daily (1 tablet thrice a day, for 6 weeks), started at the same age of 26, was reduced to 150 mg (<3 weeks) and provided mild pain relief but was stopped due to auditory hallucinations. Tetrabenazine and benzodiazepines (specific dosages and duration not obtained) were administered without significant improvement. Wrist splints restricted mobility without a significant benefit. To mitigate bruxism, the patient initially used dental splints, which proved ineffective. Subsequently, botulinum toxin injections were initiated at the age of 26 (15 units into each masseter muscle every 3–4 months), which provided significant improvement. Intermittent dysphagia was managed by a speech therapist.

Pain management involved paracetamol, anti‐inflammatories, and neuropathic pain medications. Pregabalin, started at the age of 27, was tried up to 25 mg twice daily for 3 months but was then stopped. Gabapentin (100 mg nightly for 2 months) reduced hand pain but required dosage adjustment (taken only occasionally) due to drowsiness and headaches. Carbamazepine was suggested but not initiated.

## Literature Review

We conducted a systematic review according to PRISMA guidelines,^10^ including articles on PubMed, Embase, Google Scholar, Web of Science, and ProQuest. The search covered literature published from 1994 to 2023 and was last updated on March 14, 2024. We searched for all cases diagnosed with Partington syndrome *or* diagnosed with any mutation in the *ARX* gene *and* exhibiting hand dystonia *and* discussing the management of dystonia. We used the following search terms, either as Free terms, MeSH terms, or Emtree descriptors: “Partington syndrome,” “PRTS,” “X‐linked mental retardation,” “genetic disease,” “*ARX*,” “dystonia,” “dystonic disorders,” and “treatment.” Table 1 (search strategies and number of results for different databases) provides an overview of the search strategies conducted on March 14, 2024, and the number of results. The title and abstract of the articles were then analyzed for relevance. All articles that made any reference to Partington syndrome or ARX mutation with hand dystonia were considered. Those found to be relevant were vetted against the inclusion criteria. The selection process was carried out using a data extraction grid based on the PICO (population, intervention, comparison, outcomes) framework and the specific inclusion criteria in Table 2.

**TABLE 1:** Search strategies and number of results for different databases

Search strategy	Strategy	Number of results
PubMed	(“Partington X‐linked mental retardation syndrome”[Supplementary Concept] OR “Partington syndrome”[tiab:~9] OR ((“Genetic Diseases, X‐Linked”[Mesh:NoExp] OR “Mental Retardation, X‐Linked”[Mesh:NoExp]) AND ARX) OR (“X‐Linked”[tiab] AND (“Mental Retardation*”[tiab] OR “genetic disease*”[tiab]) AND ARX) OR PRTS) AND (“Dystonia”[Mesh:NoExp] OR “Dystonic Disorders”[Mesh:NoExp] OR dystonia[tiab] OR dystonic[tiab])	21
Embase	((Partington NEAR/9 syndrome):ab,ti,kw OR ((“X linked mental retardation”/de OR “X chromosome linked disorder”/de) AND ARX) OR (“X‐Linked”:ab,ti,kw AND (“Mental Retardation*” OR “genetic disease*”):ab,ti,kw AND ARX) OR PRTS) AND (“dystonia”/de OR “dystonic disorder”/exp OR (dystonia OR dystonic):ab,ti,kw)	26
Google scholar	“Partington syndrome”|“Partington X”|“X‐Linked Mental Retardation” ARX dystonia|dystonic therapy|treatment	Limit at 235
Web of science	((Partington NEAR/9 syndrome) OR (“X‐Linked” AND (“Mental Retardation*” OR “genetic disease*”) AND ARX) OR PRTS) AND (dystonia OR dystonic)	27
ProQuest	NOFT((Partington NEAR/9 syndrome) OR (“X‐Linked” AND (“Mental Retardation*” OR “genetic disease*”) AND ARX)) AND NOFT(dystonia OR dystonic)	26

**TABLE 2:** Inclusion criteria

Articles describing patients diagnosed with Partington syndrome **OR** any ARX mutation
**AND** presenting hand dystonia
**AND** where the management of dystonia is discussed

The database search yielded 335 records, of which 19 papers were found relevant, but only 3 met all the inclusion criteria. Six studies did not describe hand dystonia,^2^, ^11^, ^12^, ^13^, ^14^, ^15^ and/or 14 studies did not discuss the management of dystonia.^2^, ^7^, ^9^, ^11^, ^13^, ^15^, ^16^, ^17^, ^18^, ^19^, ^20^, ^21^, ^22^, ^23^ Two of these studies discussed a therapeutic intervention for dystonia, but upon careful reading, there was no diagnosis of Partington syndrome and no description of hand dystonia, only generalized dystonia that was most severe in the neck and arms,^12^ with the other study describing “dystonia and spasticity” without specifying the localization.^14^

Three studies presented in Table 3 (treatment effects on hand dystonia in published literature, featuring also our 2 cases) met inclusion criteria and present cases consistent with Partington syndrome, yet they significantly differ in patient clinical presentations (eg, age range, extent of dystonia, associated symptoms, and genetic mutations). The dystonia managements reported in the few studies also diverge between the cases. The patient in study A^24^ was treated with propranolol, gabapentin, and haloperidol, whereas the patient in study B^25^ was on l‐dopa and baclofen. Study C^8^ mentioned only physiotherapy management. In all 3 studies, the details of these treatments are severely limited, lacking an objective evaluation of their effects on dystonic symptoms. Therapeutic protocols are merely mentioned, with no specifics on dosage, mode of administration, treatment duration, patient adherence, adverse effects, or final outcomes.

**TABLE 3:** Treatment effects on hand dystonia in published literature, also featuring our 2 cases

Study	Localization of dystonia	Treatment	Dystonia improvement
Arvio et al.^24^ (study A)	Hands, toes and feet, dystonic head nodding.	Propranolol, gabapentin, haloperidol, escitalopram, and levodopa (l‐dopa)/carbidopa.	Propranolol and gabapentin: no response. Haloperidol: worsening of symptoms. Escitalopram: relief for myokymias and nausea/redness attacks. l‐Dopa/carbidopa: mild improvement in bradykinesia and tremor was followed by a gradual decrease in the effect.
Breen et al.^25^ (study B)	Bilateral hand dystonia.	Levodopa, baclofen, and botulinum toxin injections.	l‐Dopa and baclofen: symptomatic benefits. Botulinum injections: effects not specified.
Frints et al.^8^ (study C)	Case 1: hands (with flexion of the palms and extension of the fingers), minor dystonic movements in the face. Case 2: hands (with flexed‐hand posture when grasping).	Physiotherapy.	No improvement.
Case 1 (our study)	Bilateral dystonic movements of the hands (involuntary wrist flexion at rest which disappeared during movement).	400‐mg l‐dopa for 2 months, then 800‐mg l‐dopa for 3 months.	Improvement in dystonia severity at 800 mg per day.
Case 2 (our study)	Bilateral hand dystonia (upper‐limb extensions, flexion of the digits, pronation of the wrists), jaw dystonia (leading to bruxism), foot dystonia.	Hand dystonia: trihexyphenidyl 2 and 4 mg, l‐dopa 300 mg (100 mg thrice a day), and then 150 mg (50 mg thrice a day) tetrabenazine, benzodiazepine, splints for the wrists and hands.	Trihexiphenidyl: no effect. l‐Dopa: pain relief but no effect on dystonia.
			Tetrabenazine and benzodiazepine: no significant effect on dystonia. Splints for the wrists: no significant improvements.

## Discussion

Our 2 case studies contribute to the clinical understanding of Partington syndrome. Despite differences in age and clinical manifestation, both cases exhibit focal hand dystonia, *ARX* mutations, intellectual disability, and dystonic tremor. Additional features include motor and visuospatial dyspraxia and bucco‐linguo‐facial apraxia (case 1), and ideomotor, ideational, and oculomotor apraxia (case 2). These signs and symptoms align with the original description,^1^ although studies report broader phenotypes, including mood disorders, epilepsy, limb‐kinetic apraxia, and dystonia affecting the face, larynx, and feet.^1^, ^5^, ^6^, ^7^, ^8^, ^9^, ^18^, ^19^, ^20^, ^24^, ^26^ Another striking difference compared to the original description is the progressive worsening of the hand dystonia in the second decade. Therefore, the quoted “nonprogressive dystonia” by Partington^1^ is not correct.

The *ARX* gene is crucial for GABAergic neuron development and function,^27^ with expression in motor‐related brain regions like the basal ganglia,^28^ which explains the motor phenotypes of the c.429_452dup24 mutation. Based on the knowledge that basal ganglia are affected, and especially because dopaminergic trials are regularly performed in the setting of pediatric dystonia, dopamine emerged as a rational therapeutic option to investigate. The l‐dopa trial resulted in only slight to mild improvements in dystonia in case 1 and pain relief in case 2, with both patients discontinuing due to side effects or treatment burden. Previous studies report limited l‐dopa efficacy for dystonia in Partington syndrome,^25^ and its use lacks strong evidence. These results may be explained by the current uncertainty regarding the pathophysiology of Partington syndrome, particularly whether dopaminergic dysfunction is involved. The blinded l‐dopa trial in case 1 was initiated after a reported “symptomatic benefit” of l‐dopa combined with baclofen in Breen et al.'s case.^25^ This was conducted prior to the publication by Lizarraga et al.,^29^ which suggests a cortical GABAergic dysfunction underlying abnormal hand movements in ARX mutation. Although an l‐dopa trial is mandatory in children developing dystonia (particularly anyone with suspected dopa‐responsive dystonia), its benefit for Partington patients remains unclear.^30^

Partington syndrome is classified as “complex dystonia” due to its combination of movement and cognitive symptoms.^31^ The typical hand dystonia is a focal dystonia,^32^ for which botulinum toxin usually is a first‐line treatment.^33^, ^34^ However, there is no clear benefit of this strategy in Partington in the few included studies, although it has been used.^25^ Botulinum toxin treatment can also be challenging due to the presence of ideomotor, ideational, or especially limb‐kinetic apraxia in these patients, which often interact with dystonia and can make the indication for treatment with botulinum toxin injections more uncertain, as impairment of fine motor coordination may be predominantly due to the apraxia and as too much toxin‐induced weakness can preclude rehabilitation.

Oral medications resulted in poor outcomes, as seen in our review^24^ and case 2 (who tried l‐dopa, trihexyphenidyl, tetrabenazine, and benzodiazepines without significant improvement). Gabapentin helped with pain but caused side effects. However, Breen et al.^25^ mention that baclofen, an anti‐spastic agent often tried in childhood‐onset dystonia,^30^ was effective in a young 12‐year‐old boy, in association with l‐dopa. It is important to note that these results should be interpreted with caution and may be insufficient to draw a definitive conclusion regarding the efficacy or inefficacy of either treatment due to the limited data available.

Additionally, unpublished discussions with the author revealed that in Curie's study of 27 patients with the dup24 mutation,^9^ 74% had focal dystonia, mild to moderate in 55% and severe in 19%. Various treatments (eg, anticholinergics, l‐dopa, and neuroleptics) were tried. Unfortunately, these treatments provided only limited or temporary benefits, and specific details about their use were not included in the published paper.

Physical rehabilitation, including stretching, strengthening, and splinting, is commonly used for hand dystonia, but there are no established guidelines or documented outcomes in Partington syndrome.^35^

“Geste antagoniste,” also called “sensory tricks,” are voluntary maneuvers that reduce the severity of abnormal postures and may completely, though transiently, alleviate posturing and associated disability.^36^ Except these well‐known sensory tricks, visual and auditory tricks have been rarely reported, improving posturing in a case of camptocormia^37^ or improving generalized dystonia in a DYT‐TOR1A case upon playing the piano.^38^ Another article demonstrated no improvement in dystonia on altered auditory and tactile feedback.^39^ In case 1, hand dystonia improved significantly when playing basketball, the hand opening before the ball touching it, probably due to visual and/or auditory cueing (Video 4).

In conclusion, no consistently effective treatment for Partington syndrome–related dystonia has been identified, particularly due to the syndrome's rarity and variability. l‐Dopa provided minimal benefits but was limited by side effects. Further studies are needed to explore therapeutic options, determine the syndrome's pathophysiology, and improve management for patients with *ARX* mutations in general. Descriptions of new cases and long‐term follow‐ups are essential to better understand this complex disorder.

## Author Roles

**1. Research project:** A. Conception, B. Organization, C. Execution.

**2. Manuscript preparation:** A. Writing of the first draft, B. Review and critique.

E.P.: 1A, 1B, 1C, 2A

A.A.: 1A, 1C, 2A

S.C.C.: 1A, 1C, 2A

A.Z.: 1C, 2B

M.R.‐L.: 1C, 2B

D.P.B.: 1C, 2B

F.Z.‐G.: 1C, 2B

F.D.: 2A, 2B

A.C.: 2B

D.W.: 1A, 2A

V.S.C.F.: 2B

S.D.: 2B

J.F.: 1A, 2B

J.F.B.: 1A, 1B, 1C, 2B

## Disclosures

**Ethical Compliance Statement**: Written consent was obtained from both patients, and all identifying details were appropriately anonymized to ensure privacy. We confirm that we have reviewed the journal's ethical publication guidelines and affirm that this work is consistent with those guidelines. We also confirm that the approval of an institutional review board was not required for this work.

**Funding Sources and Conflicts of Interest**: This research received no specific funding. The authors declare that there are no conflicts of interest related to this work.

**Financial Disclosures for the Previous 12 Months**: The following disclosures, unrelated to this work, are reported for the past 12 months: E.P. declares that there are no additional disclosures to report. A.A. receives partial congress fees from Merz. S.C.C. declares that there are no additional disclosures to report. A.Z. declares that there are no additional disclosures to report. M.R.‐L. declares that there are no additional disclosures to report. D.P.B. received honoraria from Bial Pharma UK Ltd and Springer Nature Limited. F.Z.‐G. declares that there are no additional disclosures to report. F.D. declares that there are no additional disclosures to report. A.C. declares that there are no additional disclosures to report. W.D. declares that there are no additional disclosures to report. V.S.C.F. receives a salary from NSW Health, has received unrestricted research grants from the Michael J. Fox Foundation, AbbVie, and Merz, and receives royalties from Health Press Ltd and Taylor and Francis Group LLC. S.D. received honorary and speaker fees from AbbVie and Seqirus. J.F. serves as a consultant on the advisory board of Roche and Biogen. J.F.B. received consultant honorary and/or speaker's honorary and/or partial congress fees from AbbVie, Medtronic, Spirig, Bial, Zambon, and Merz.

## Supporting information


**Appendix S1.** Methods and results of Partington's assessment under levodopa. Part A: blinded assessment by 3 independent movement disorders experts from 3 movement disorders centers (United Kingdom, Spain, and Switzerland). Part B: unblinded assessment by our neuropsychologist at baseline and then at 800‐mg levodopa per day.

## Data Availability

The data that support the findings of this study are available from the corresponding author upon reasonable request.
