# Supplementary material for: Managing Dystonia in Partington Syndrome
Source: Mov Disord Clin Pract. 2025 Sep 30;13(2):533–40. doi: 10.1002/mdc3.70356 (PMC13020569; doi:10.1002/mdc3.70356)
Supplement: Supplementary file 6 — Appendix S1. Methods and results of Partington's assessment under levodopa. Part A: blinded assessment by 3 independent movement disorders experts from 3 movement disorders centers (United Kingdom, Spain, and Switzerland). Part B: unblinded assessment by our neuropsychologist at baseline and then at 800‐mg levodopa per day. [file MDC3-13-533-s001.pdf]

SUPPLEMENTAL APPENDIX

Appendix A: Methods and Results of Partington's assessment under levodopa

Methods

A specific video protocol was developed to standardize the recording of each evaluation. The protocol included seventeen items focusing on various aspects such as the oro-buccal sphere, neck, arms, legs, writing, and posture. The patient was recorded before levodopa treatment, two months after reaching a stable dose of 400 mg per day, and three months after reaching 800 mg per day. Each session was conducted with the same clothing to minimize subjective influence and included photographs of the patient's writing. Three independent movement disorder experts from the United Kingdom, Spain, and Switzerland blindly evaluated each video and photo. They used the Burke-Fahn-Marsden (BFM) score and the Writer's Cramp Evaluation scale to objectively assess dystonia severity and subjectively identified the best and worst videos for dystonia and writing.

Unblinded neuropsychological evaluations were conducted at baseline and after reaching 800 mg of levodopa per day. Manual dexterity was assessed using the Pegboard test, and constructive praxis was evaluated with the Cerad and Rey figure tests, along with assessments of bucco-linguo-facial, gestural, and ideational praxis. This evaluation was not blinded.

Results

In red : the results that came out **worse** with increasing the levodopa dose.

In green : the results that came out **better** with increasing the levodopa dose.

Italic is baseline score.

Part A: blinded assessment by 3 independent movement disorders expert from 3 MDs centers (UK, Spain and Switzerland)

Part I: dystonia scale (BFM severity scale) – analyze based on the video

	No levodopa	**400 mg per day**	**800 mg per day**
AZ	*22*	**24.5**	**19.5**
MRL	*26.5*	26.5	**25.5**
DPB	*29.5*	**25.5**	25.5

Part II: writer's cramp scale (movement then speed scales) - analyze based on the video

	No levodopa	**400 mg per day**	**800 mg per day**
AZ	*10+1*	**8+2**	**6+2**
MRL	*12+1*	12+1	12+1
DPB	*16+1*	16+1	**12+1**

Part III: subjective assessment of the writing's quality - analyze based on the picture of the writing

	No levodopa	**400 mg per day**	**800 mg per day**
AZ	*Worst*	**Intermediate**	**Best**
MRL	*Worst*	**Intermediate**	**Best**
DPB	*Worst*	**Intermediate**	**Best**

Part IV: subjective overall assessment – as per personal opinion embedded in the experts' emails

	No levodopa	**400 mg per day**	**800 mg per day**
AZ	*Worst*	**Intermediate**	**Best**
MRL	*Worst*	**Intermediate**	**Best**
DPB	*Worst*	**Intermediate**	**Best**

Part B: unblinded assessment by our neuropsychologist at baseline, then at 800 mg levodopa per day:

	No levodopa	**800 mg per day**
Pegboard L hand	*1*	**4**
Pegboard R hand	*4*	**2**
Constructive praxia: Cerad figure	*8*	8
Constructive praxia: Rey figure: copy	*29*	**31.5**
Constructive praxia: Rey figure: time	*446 s*	**326 s**
BLF praxia	*5*	**9**
Gestural praxia	*8*	8
Ideational praxia: candle	*Partial success*	**success**
Ideational praxia: envelope	*success*	success
Ideational praxia: locker	*success*	success

23 items came green

2 items came red

9 items showed no change
